# Supplementary figures and images for: Redefining the spliceosomal introns of the sexually transmitted parasite Trichomonas vaginalis and its close relative in columbid birds
Source: PLoS Pathog. 2025 Jul 23;21(7):e1013282. doi: 10.1371/journal.ppat.1013282 (PMC12316389; doi:10.1371/journal.ppat.1013282)

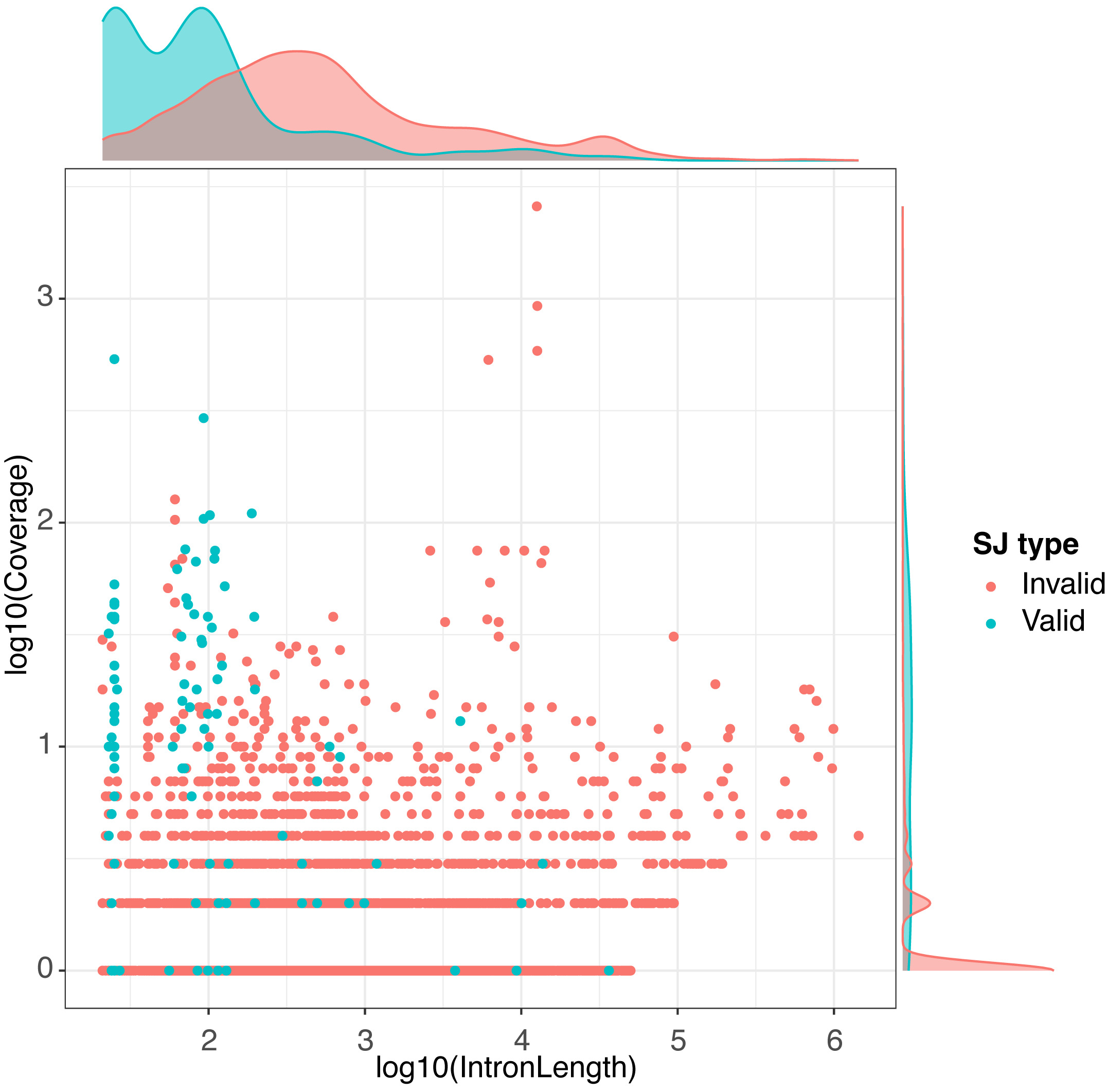

Supplement: S1 Fig — Coverage and length (log10-based) for each SJ are shown in the “Y” and “X” axes, respectively. SJs were classified as invalid and valid by filtering those mapping to genomic regions lacking the degenerated intron motifs needed by the spliceosomal machinery. (TIF) [file ppat.1013282.s001.tif]

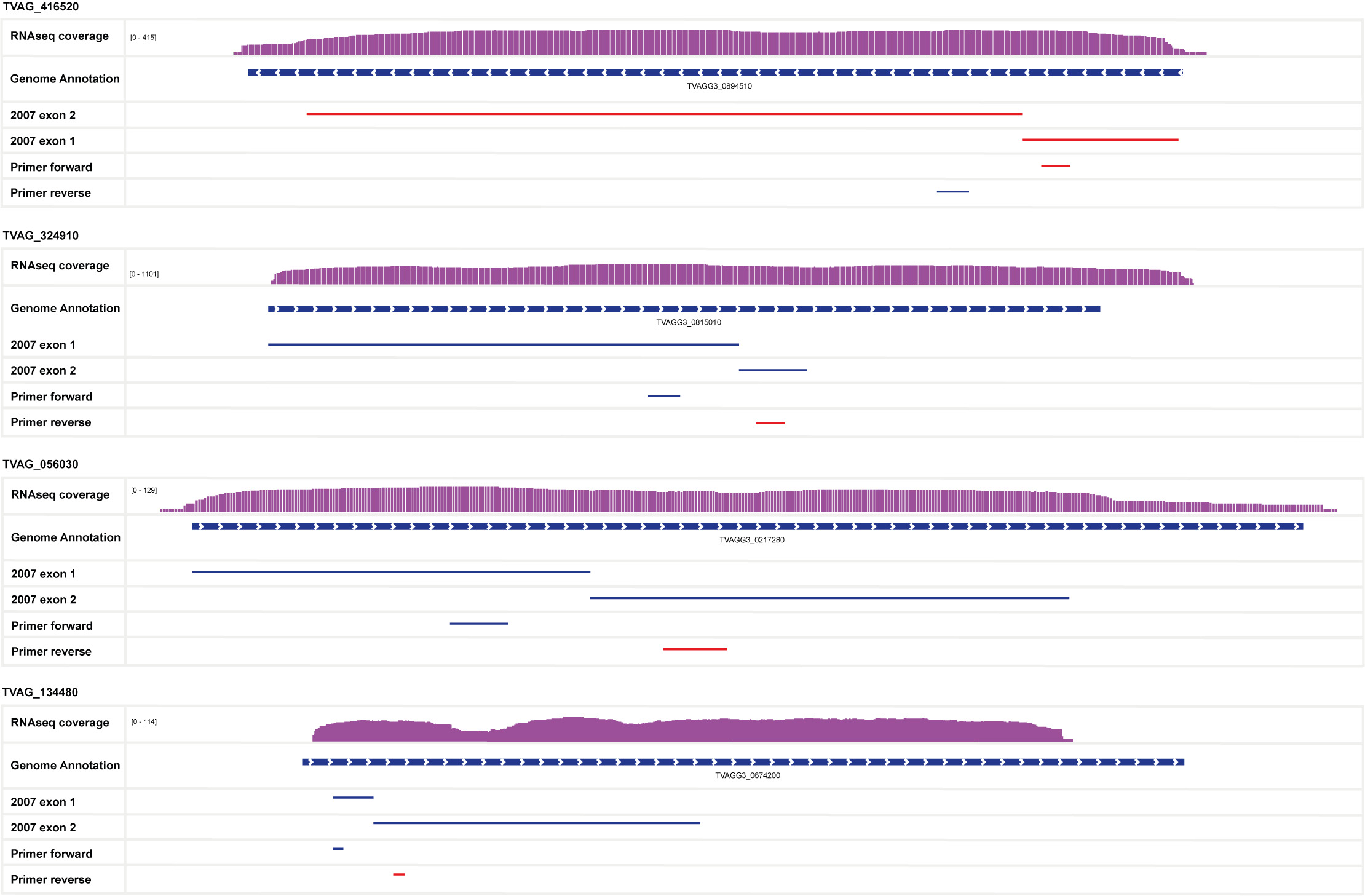

Supplement: S2 Fig — The RNAseq coverage (violet) mapped to the new genome and the new genome annotation are shown in the first and second panels, respectively (top to bottom). The exons predicted by Vanacova et al. [12], Wang et al. [11], and the first genome annotation [8] are shown in the following panels (3,4). The primers used for their validation by Wang et al. [11] are shown in the bottom panels (5,6). (TIF) [file ppat.1013282.s002.tif]

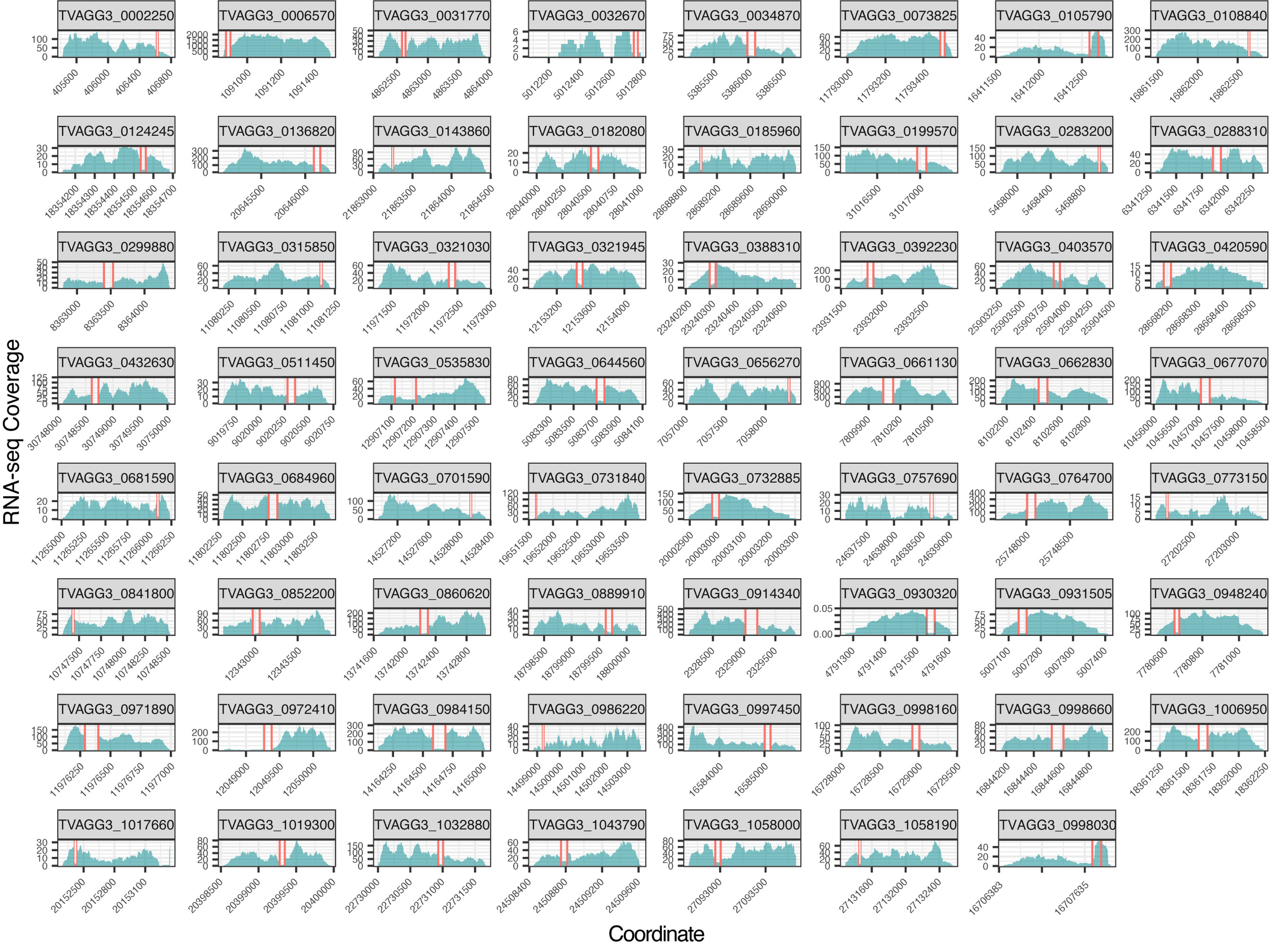

Supplement: S3 Fig — The intron sequences are delimited by vertical red lines, the RNAseq coverage is shown in cyan (y-axis), and the transcript length is indicated in the x-axis. (TIF) [file ppat.1013282.s003.tif]

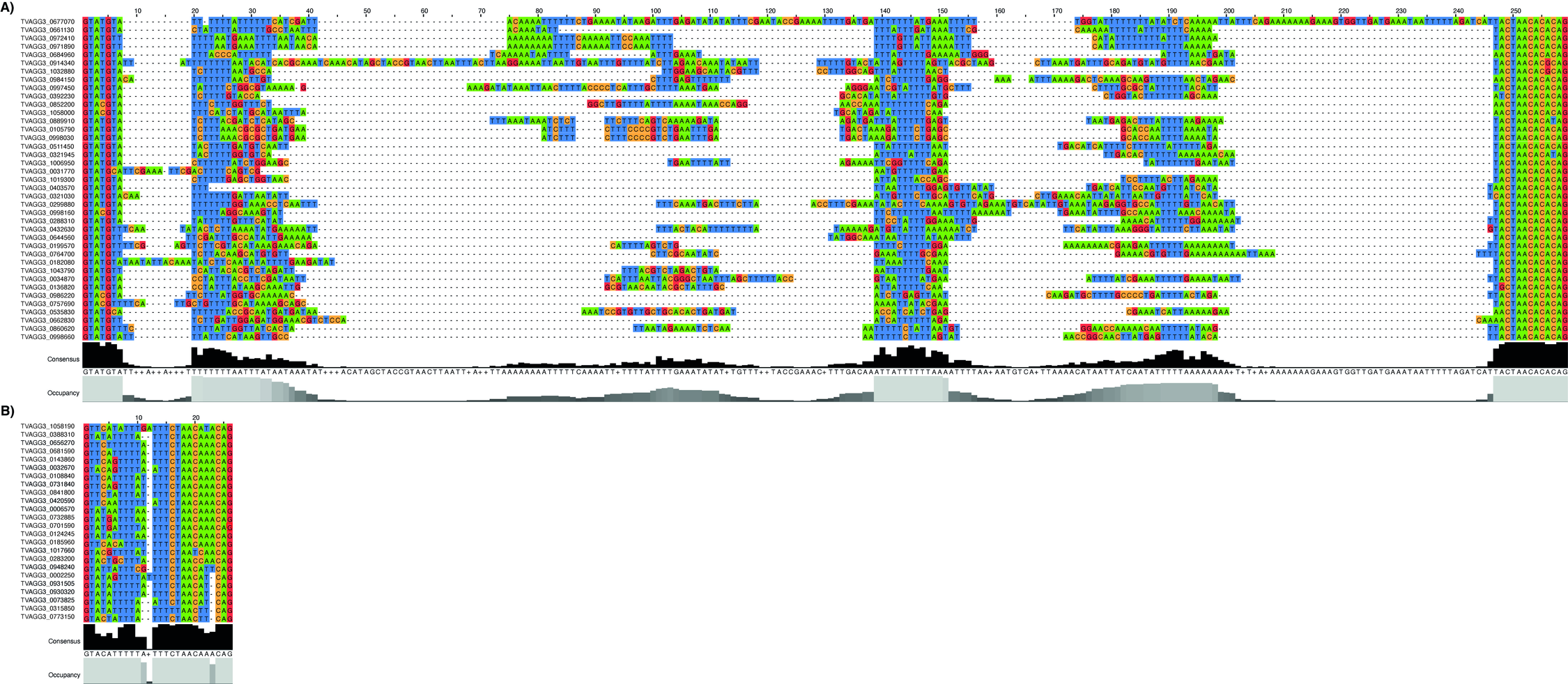

Supplement: S4 Fig — The upper panel shows the MSA for the type A introns (length from 56-196 nucleotides), and the lower panel shows type B introns. (TIF) [file ppat.1013282.s004.tif]
